# Aluminum plasmonic nanoshielding in ultraviolet inactivation of bacteria

**DOI:** 10.1038/s41598-017-08593-8

**Published:** 2017-08-22

**Authors:** Jeremy N. Kunz, Dmitri V. Voronine, Weigang Lu, Zachary Liege, Ho Wai Howard Lee, Zhenrong Zhang, Marlan O. Scully

**Affiliations:** 1 Baylor University, Waco, Texas 76706 USA; 2 Texas A&M University, College Station, Texas 77843 USA

## Abstract

Ultraviolet (UV) irradiation is an effective bacterial inactivation technique with broad applications in environmental disinfection. However, biomedical applications are limited due to the low selectivity, undesired inactivation of beneficial bacteria and damage of healthy tissue. New approaches are needed for the protection of biological cells from UV radiation for the development of controlled treatment and improved biosensors. Aluminum plasmonics offers attractive opportunities for the control of light-matter interactions in the UV range, which have not yet been explored in microbiology. Here, we investigate the effects of aluminum nanoparticles (Al NPs) prepared by sonication of aluminum foil on the UVC inactivation of *E*. *coli* bacteria and demonstrate a new radiation protection mechanism via plasmonic nanoshielding. We observe direct interaction of the bacterial cells with Al NPs and elucidate the nanoshielding mechanism via UV plasmonic resonance and nanotailing effects. Concentration and wavelength dependence studies reveal the role and range of control parameters for regulating the radiation dosage to achieve effective UVC protection. Our results provide a step towards developing improved radiation-based bacterial treatments.

## Introduction

Bacterial inactivation has recently received much attention due to the rising concern of antibiotic resistance^1^. Many alternative bacterial inactivation techniques have been developed, including the promising light-induced photo-inactivation methods such as photodynamic^2–6^ and photothermal^7–11^ treatments. The former relies on the interaction of light at frequencies typically in the visible and near-infrared range with photosensitizers which produce reactive species. The latter is based on the local heat generated using visible or near-infrared light interacting with plasmonic nanostructures made of Ag, Au and other metals. The advantages of these techniques are high efficiencies, robustness to bacterial resistance, and localized treatments. However, the disadvantages are the limited selectivity and intrinsic toxicity of the metal nanoparticles to beneficial bacteria and healthy tissue^12, 13^. This requires developing complex photosensitizer delivery and nanoparticle functionalization methods.

Ultraviolet (UV) radiation has also been used for bacterial inactivation finding many applications such as the treatment of corneal^14^ and wound infections^15, 16^, medical device^17^, water^18, 19^ and air^20–22^ disinfection, and many others. UV radiation inactivates bacteria by damaging biomolecules such as DNA and proteins^22–26^. However, it does not discriminate between beneficial and pathogenic bacteria or healthy tissues. In order to develop selective bacterial UV disinfection treatments, it is necessary to improve UV protection techniques which could be used to counteract the UV inactivation for selected targets. Different mechanisms are responsible for the radiation damage in the three different spectral ranges: UVA (320–400 nm), UVB (290–320 nm), and UVC (100–290 nm). The UVC range was found to be the most efficient for the bacterial inactivation^24, 26^. Ag and metal oxide (e.g. ZnO, TiO_2_) nanoparticles were previously used in sunscreens for the protection against UVA and UVB radiation^27–29^. The nanoparticles are more efficient UV protective agents than molecules due to their UV absorption and scattering properties as opposed to the mere molecular absorption. However, there is still a lack of studies on the efficiency of nanoparticle-based bacterial protection techniques against UVC. For example, there has been evidence for self-production of the UVC “sunscreen” based on Fe(III) compounds by some bacteria^30^. Also, the microparticle-based UVC protection of bacteria in wastewater was investigated^17, 32^. In the former case, only a few types of bacteria have the ability to synthesize Fe-based compounds. In the latter case, the disadvantages of the large size (>20 μm in ref. 32 and >60 μm in ref. 31) microparticles and low protection efficiency (<1%) limit the practical applications. To overcome these disadvantages it is important to reduce the size of the UV shielding particles and to improve their protection efficiency. Aluminum plasmonics provides attractive opportunities for the control of light-matter interactions in the UVC range.

Plasmon-enhanced light-matter interactions have been intensively investigated using traditional coinage metals such as Ag and Au whose plasmonic resonances are in the visible spectral range. Al is an alternative, inexpensive plasmonic material with broad tunability from near-infrared to the deep UV^33, 34^. Therefore, Al nanostructures may be used to extend plasmonic applications to biological systems with electronic UV resonances. This leads to interesting applications such as UV surface-enhanced fluorescence^35–38^ and Raman^39, 40^ bio-sensing and bio-imaging, plasmon enhanced light harvesting^41, 42^ and strong quantum plasmonics effects^43^. However, antimicrobial applications of the aluminum plasmonics have not yet been explored.

**Figure 1:**
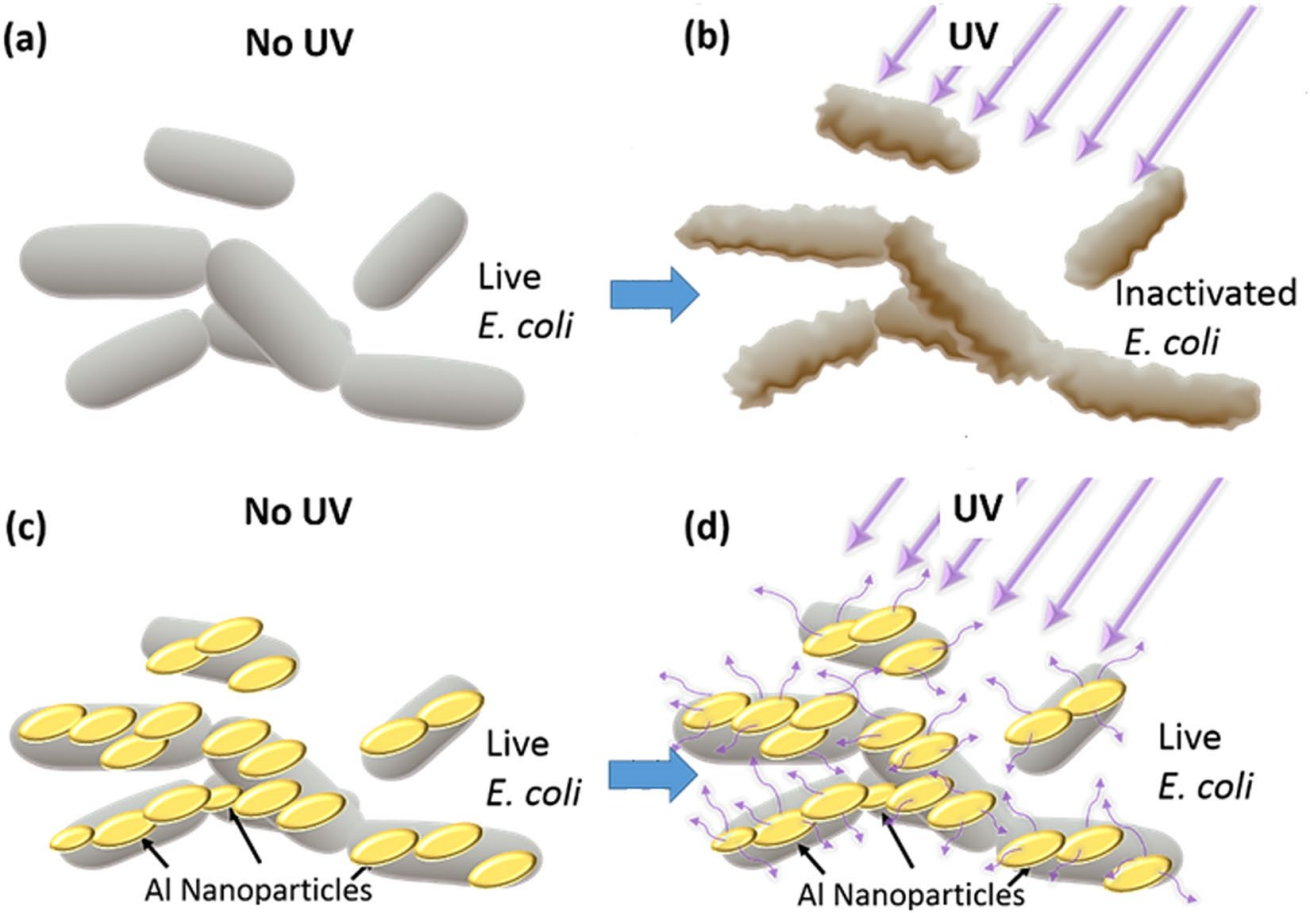
Shielding of *E*. *coli* bacteria from UV radiation using plasmonic aluminum nanoparticles (Al NPs). Schematic of the UV radiation effects on *E*. *coli* without (**a**,**b**) and with (**c**,**d**) Al NPs. After exposure to UV radiation, the unprotected bacteria are inactivated (**b**) but the protected bacteria are unharmed (**d**). The nanoshielding is achieved via absorption and scattering of UV radiation by the plasmonic Al NPs interacting with the bacterial cells.

Here we investigate the effects of aluminum nanoparticles (Al NPs) on the UV inactivation of *Escherichia coli* (*E*. *coli*) K12 bacteria and demonstrate a new UV protection mechanism via plasmonic nanoshielding. Figure 1 schematically depicts two different cases: (i) *E*. *coli* exposed to UV radiation without Al NPs (Fig. 1a and b), and (ii) *E*. *coli* exposed to UV radiation with Al NPs present in the solution (Fig. 1c and d). In the case (ii) the direct contact between *E*. *coli* and Al NPs is possible, and UV shielding via absorption and scattering of UV radiation may apply to both freely-suspended and nanoparticle-associated bacteria. Our results show that UV nanoshielding has no effect on the freely-suspended bacteria and is primarily achieved due to the direct interaction between *E*. *coli* and Al NPs.

## Materials and Methods

*E*. *coli* K12 bacteria was obtained in slant tubes from Carolina Biological. *E*. *coli* was removed from the slant tubes using a sterile inoculation loop and placed in a 10 ml Lysogeny broth (LB). The broth was then placed in an incubating shaker (Fisher Scientific) at 37 °C and 180 rpm for 24 hours to obtain a final concentration of ~1.5 × 10^9^ colony forming units (CFU) per ml. The liquid broth culture was stored at 4 °C until use.

**Figure 2:**
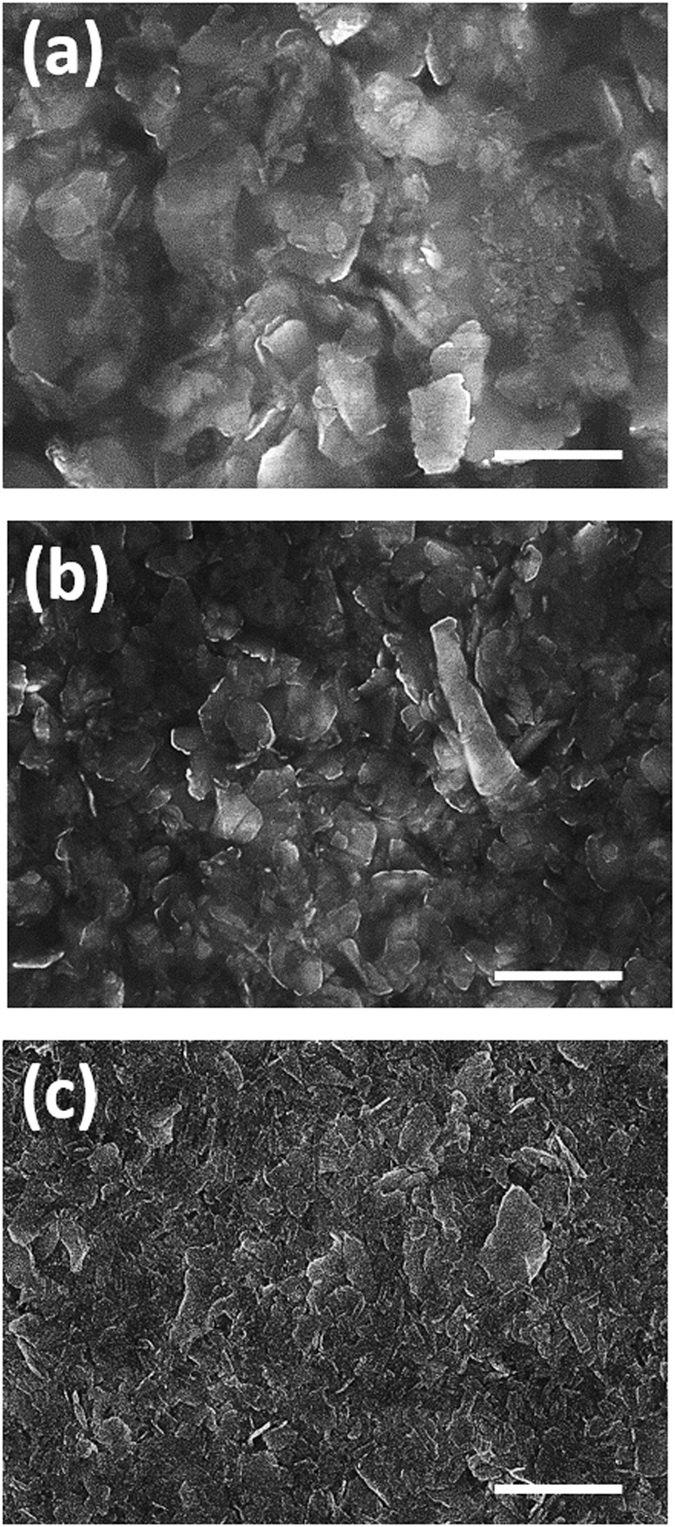
Scanning electron microscopy (SEM) images of the aluminum particles with different sizes. SEM images of the precipitated Al particles separated at 8000 rpm (**a**), 12000 rpm (**b**), and 20000 rpm (**c**). Large Al microparticles are present in all three cases but the number of large particles is reduced with the increased rpm. The scale bar is 2 μm.

**Figure 3:**
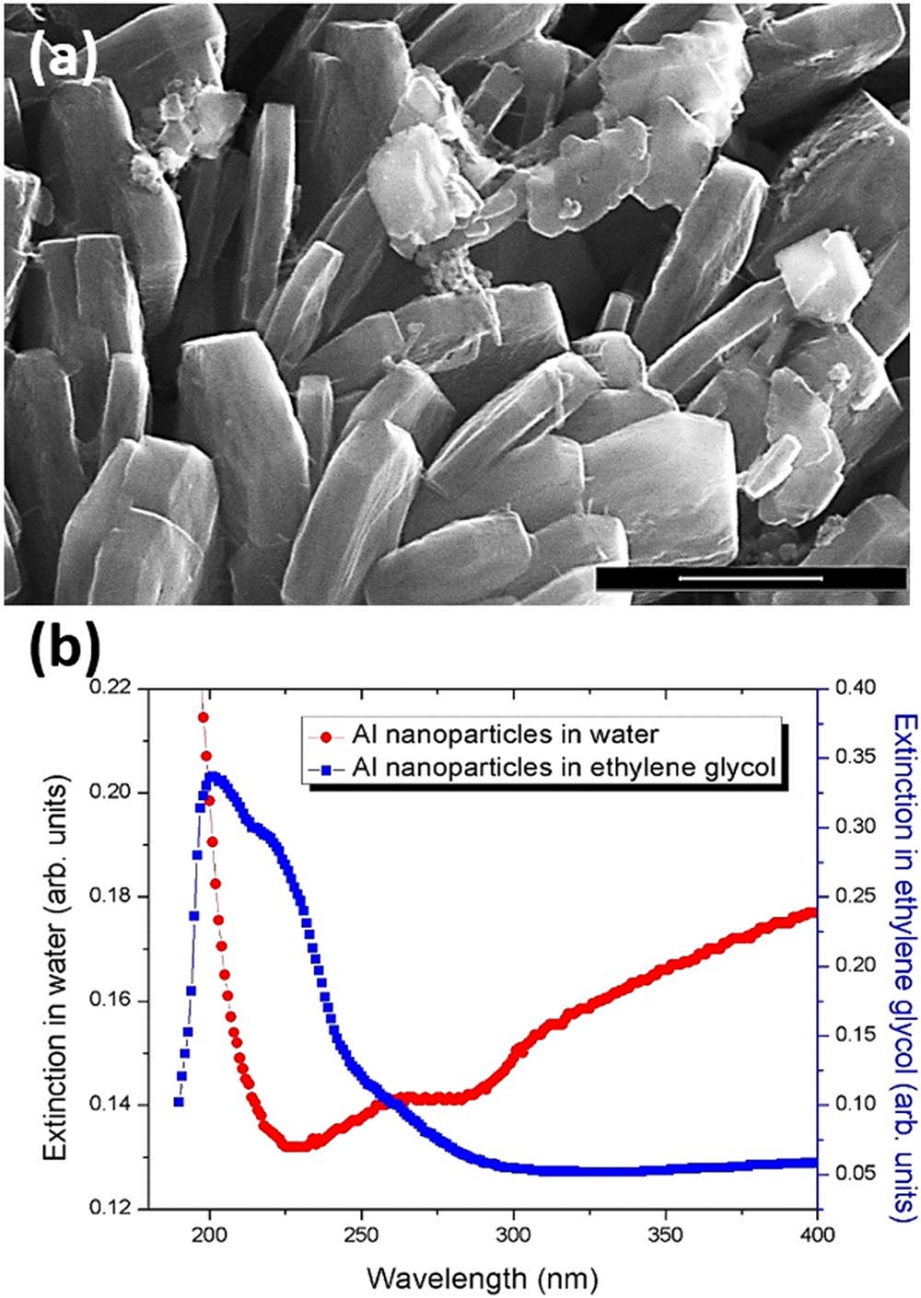
Characterization of aluminum nanoparticles (Al NPs). (**a**) Scanning electron microscope (SEM) image of the disk-like Al NPs. The scale bar is 500 nm. (**b**) Extinction spectra of Al NPs in ethylene glycol (blue squares) and after washing in water (red circles).

To fabricate the Al NPs, 0.16 g of Al foil (Reynolds Wrap®) was cut into 2 mm × 2 mm pieces and put into 100 ml ethylene glycol. The mixture was put into Branson 2510 ultrasonic cleaner (Branson Ultrasonics Danbury, CT, USA) for ultrasonic milling. During the sonication, it was observed that Al foil gradually fragmented to smaller pieces and the color of the mixture solution slowly changed from a colorless to grayish black. After 20 hours of sonication the mixture was left for an hour to settle. The upper layer from this solution was centrifuged at 2000 rpm (Beckman Coulter Optima XPN 1000) for 10 minutes to remove large particles. Al NPs of different sizes were separated at 8000, 12000, 20000 and 25000 rpm for 20 min, respectively. Scanning electron microscopy (SEM) was used to determine the average size of these Al particles to be >2 μm, ~2 μm, 1–2 μm, and <1 μm, respectively. Figure 2 shows SEM images of the Al particles precipitated from the first three solutions which all have large microparticles with the sizes of more than 1 μm. The effects of such micrometer size particles on the UV inactivation of bacteria were previously extensively studied and UV shielding was observed^31, 32^. Here we investigated the properties of the nanometer size Al NPs with the different UV shielding mechanism due to their aluminum plasmonics rather than the large size effect. Therefore, we discarded the first three solutions and used the solution obtained at 25000 rpm which mostly contained small nanometer sized Al NPs shown in Fig. 3a.

Al NPs were washed four times before adding to the *E*. *coli* solution by centrifugation in deionized water at 3700 rpm for 5 min. The fifth and final washing was similarly performed in deionized water at 3700 rpm for 10 min. Supernatant was drained and Al NPs were stored at room temperature in deionized water before the addition to the *E*. *coli* solution. The sonication resulted in the suspension of disk-like Al NPs with ~200–1000 nm diameter and ~50–200 nm thickness (Fig. 3a and b).

The sample solution was prepared by combining 25% by volume *E*. *coli* liquid culture (1.5 × 10^9^ CFU/ml) in LB broth with 25% Al NP solution (18.8 mg/ml); the remaining 50% of the solution was deionized water. 5 µl of the total sample solution was placed on nutrient agar in a petri dish and circularly spread to a diameter of approximately 1.5 cm using a sterilized spatula. The sample was then placed in the beam path of a Xenon lamp (Spectral Products, ASB-XE-175) using a monochromator (Spectral Products, CM110) to select the desired UV spectral range. Illumination was performed for 5, 10, 20, and 30 minutes resulting in the UV doses of 9, 18, 36 and 54 mJ/cm^2^, respectively. The samples were then incubated at 37 °C for 24 hours. Each set of measurements was repeated six to eight times with qualitatively similar results.

For the SEM measurements, the *E*. *coli* bacterial cells (with and without nanoparticles) were fixed with 2.5% glutaraldehyde in 0.06 M phosphate buffer (pH 7.2) for 90 minutes at room temperature and then washed 4 times 10 minutes each in the buffer solution. The samples were then dehydrated using a graded series of ethanol for 2 times 10 minutes at each step (50%, 70%, 90%, and 100%). After critical point drying (EM CPD300, Leica Microsystems, Wetzlar, Germany) the samples were mounted on stubs and sputter coated (EM ACE 600, Leica Microsystems) with approximately 15 nm of carbon. SEM measurements were performed in a Versa 3D scanning electron microscope (FEI, Hillsboro, OR, USA). Energy-dispersive X-ray spectroscopy (EDS) was performed with an Octane Pro Silicon Drift Detector (EDAX, Mahawah, NJ, USA).

## Results

**Figure 4:**
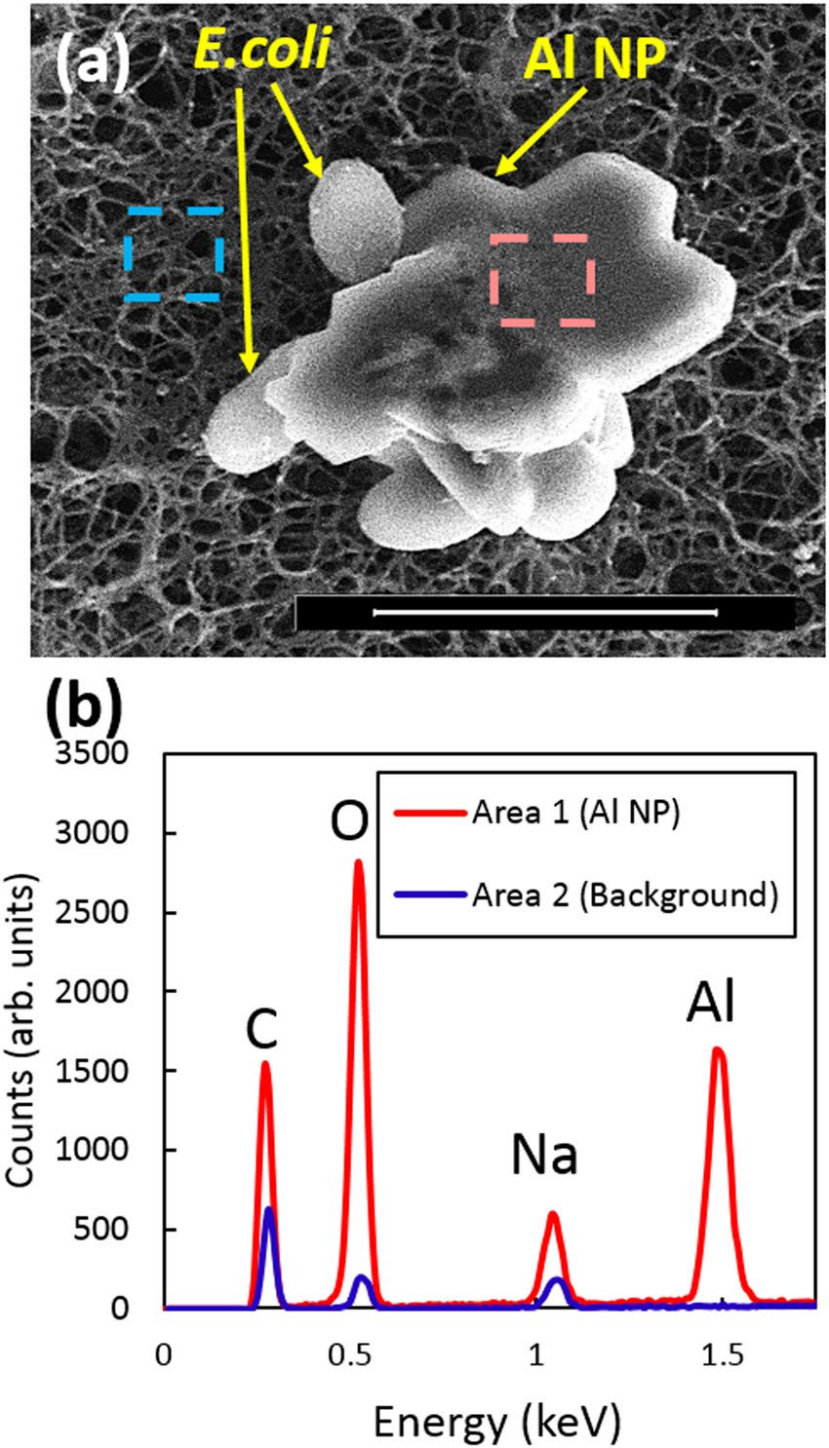
Characterization of *E*. *coli* bacteria shielded by aluminum nanoparticles (Al NPs). (**a**) SEM image of the *E*. *coli* bacterial cells shielded by Al NPs. The scale bar is 2 μm. (**b**) EDS spectra show elemental compositions of the areas on the Al NPs (red) and on the substrate (blue) corresponding to the highlighted dashed red and blue areas in (**a**), respectively, confirming the physical “shielding” of *E*. *coli* by Al NPs.

SEM images show Al NPs (Fig. 3a) and *E*. *coli* shielded by Al NPs (Fig. 4a). Most of the Al NP disks in Fig. 3a appear standing up and several disks lying flat, which clearly shows that the shapes of the Al NPs are flat irregular disks. The diameters of the Al NPs are ~200–1000 nm and the thicknesses are ~50–200 nm. After mixing Al NPs with *E*. *coli*, their aggregates are formed (Fig. 4a). Energy-dispersive spectroscopy (EDS) analysis was performed to confirm the elemental composition of the Al NPs attached to the bacteria. Figure 4b shows the presence of the Al and O elements in the nanoparticle-bacteria aggregate (corresponding to the red dashed square in Fig. 4a) and their absence in the substrate (corresponding to the blue dashed square in Fig. 4a). This confirms the presence of Al_2_O_3_ on the surface of Al NPs under ambient conditions. The oxidation state of the Al NPs corresponds to the core-shell metal/oxide NPs which was confirmed using EDS. This is consistent with the reported results on Al nanoparticles^34, 44, 45^.

Figure 3b shows extinction spectra of the Al NPs in ethylene glycol (blue squares) and after washing in water (red circles). The spectra in ethylene glycol reveal strong and sharp plasmonic resonances in the range between 200 and 250 nm and a weak shoulder around 260 nm. The spectra in water were obtained by washing the Al NPs which were prepared in ethylene glycol and show a stronger peak at ~260 nm and a broad red-shifted band extending into the visible range. This spectral behavior may be attributed to the partial aggregation of small Al NPs which have a stronger UV resonance around at shorter wavelengths^35, 36^. The stronger resonances at 260 nm and at longer wavelengths are suitable for the excitation wavelengths of 254 and 280 nm used for the UV inactivation as described below.

**Figure 5:**
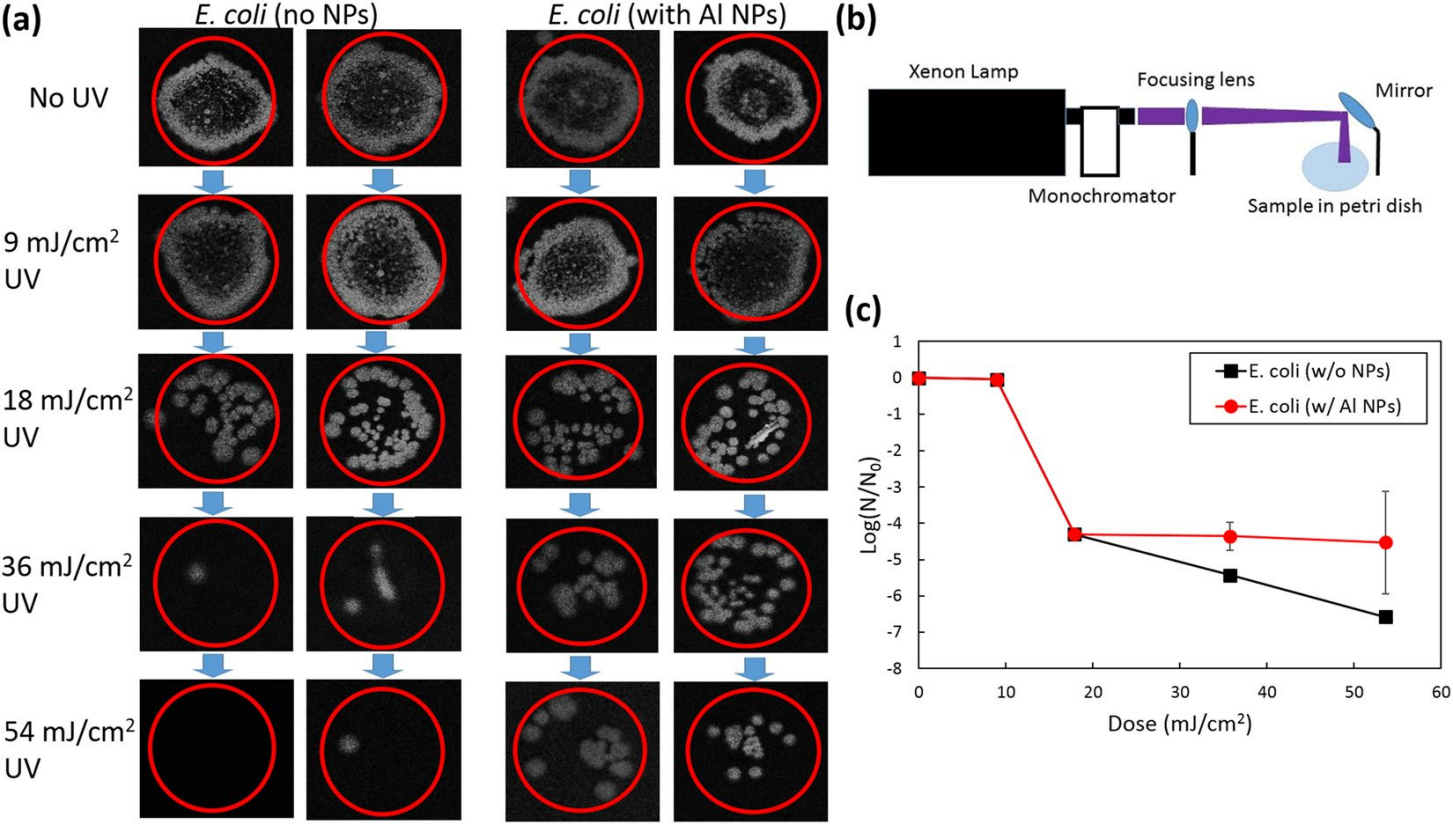
Nanoshielding effects in UV inactivation of *E*. *coli* bacteria. (**a**) Photographic images showing the culture growth of *E*. *coli* bacteria after exposure to 0, 9, 18, 36 and 54 mJ/cm^2^ dose of UV radiation at 254 nm with and without Al NPs. (**b**) Schematic of the experimental setup. (**c**) UV dose dependence of *E*. *coli* survival ratio (log(N/N_0_)) with and without Al NPs, where N_0_ and N are the numbers of the viable bacterial colony-forming units (CFUs) before and after the irradiation, respectively.

The UV nanoshielding effect is demonstrated in the bacterial viability measurements shown in Fig. 5. Figure 5b shows a simplified setup for the nanoshielding experiments which consists of a UV lamp and a monochromator for wavelength selection. The focused UV radiation is directed onto the *E*. *coli* sample with or without Al NPs placed on the surface of nutrient agar in a Petri dish. The radiation dosage was varied by changing the illumination time. After irradiation the samples were incubated for 24 hours and the resulting bacterial colony-forming units (CFUs) were counted. Figure 5a shows data from a single Petri dish with two separate irradiated sample areas highlighted by red circles. No significant *E*. *coli* colony formation after the 54 mJ/cm^2^ UV radiation dose without Al NPs was observed. However, with the addition of Al NPs, a fraction of *E*. *coli* bacteria were still able to thrive and form colonies after incubation. The UV dose-response dependence for the inactivation of *E*. *coli* with and without Al NPs is shown in Fig. 5c. The *E*. *coli* survival ratio N/N_0_ was determined by direct counting of the CFUs in the Petri dishes and by using grey value image analysis for the UV doses higher and lower than 10 mJ/cm^2^, respectively.

We also performed control measurements under identical conditions with the same concentration of Al NPs placed on a quartz slide separating Al NPs and *E*. *coli* and exposed to UV radiation (not shown). Nearly all *E*. *coli* were inactivated after the 54 mJ/cm^2^ UV irradiation regardless of the presence of Al NPs similar to the results without NPs shown in Fig. 5. These control results, in conjunction with the results showing that Al NPs preserved *E*. *coli* CFUs in contact with nanoparticles, imply that the shielding effect is due to the direct interaction of *E*. *coli* with Al NPs.

**Figure 6:**
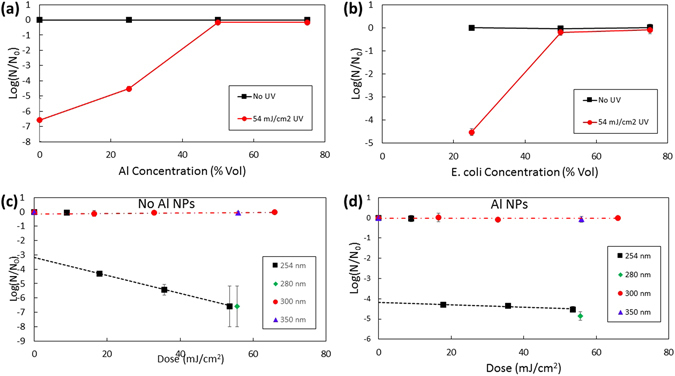
Concentration and wavelength dependence of the aluminum nanoshielding effects in bacterial inactivation. Concentration dependence of the *E*. *coli* survival ratio (log (N/N_0_)) with varying concentration of Al NPs with 25% *E*. *coli* (**a**) and with varying concentration of *E*. *coli* with 25% Al NPs (**b**) at 254 nm UV irradiation. Wavelength dependence of the *E*. *coli* survival ratio without (**c**) and with 25% (d) Al NPs at 25% *E*. *coli* concentration. Dashed and dash-dotted lines are trend lines for the measurements performed at 254 nm and 300 nm irradiation, respectively, for the doses >10 mJ/cm^2^.

The concentration dependence of the Al NPs and *E*. *coli* is shown in Fig. 6a and b, respectively. Figure 6a shows the dependence of the *E*. *coli* survival ratio log(N/N_0_) on the concentration of *E*. *coli* with 25% Al NPs in water without (black squares) and with 54 mJ/cm^2^ dose (red circles) UV radiation at 254 nm. As the Al NP concentration is increased, the shielding efficiency of *E*. *coli* bacteria from the damaging UV radiation increased (i.e. more CFUs were conserved, see Fig. 6a). The addition of increasing concentrations of Al NPs without exposure to UV radiation showed no significant change in the number of viable CFUs; therefore, Al NPs themselves do not have any toxic effects on the *E*. *coli*. The maximum protection efficiency was achieved at 50% and higher concentration of Al NPs. Figure 6b shows a similar increase of the survival ratio for the increased *E*. *coli* concentration above 50%. This indicates that a larger number of CFUs are shielded due to the increased probability of the direct interaction with the Al NPs.

The wavelength dependence of the Al NP effects on the UV irradiation of *E*. *coli* is shown in Fig. 6c and d. The survival ratios log(N/N_0_) for the UVC irradiation at 254 nm (black squares) and 280 nm (green diamonds), UVB irradiation at 300 nm (red circles), and UVA irradiation at 350 nm (blue triangles) are shown for different dosages without (Fig. 6c) and with (Fig. 6d) Al NPs. Figure 5c shows that without Al NPs both the 254 nm and 280 nm radiation was able to inactivate *E*. *coli*. In comparison, irradiation at 300 nm and 350 nm under identical conditions did not have any significant effects for the doses <80 mJ/cm^2^. When 25% of Al NPs were used, the inactivation of *E*. *coli* was suppressed at all wavelengths (Fig. 6d). The dashed and dash-dotted trend lines in Fig. 6c and d indicate the dependence of the survival ratios on the radiation dosage in the range above 10 mJ/cm^2^. The flat trend line in Fig. 6d for the case with the Al NPs shows a nanotailing effect of a weak dose dependence due to the effective shielding of the UV radiation by the Al NPs. This effect is absent in the case of the unprotected *E*. *coli* without Al NPs (Fig. 6c).

## Discussion

Size and shape of Au and Ag NPs plays an important role in the efficiency of the bacterial inactivation due to the tunability of their plasmonic resonances in the visible and NIR ranges^8–10^. These nanoparticles may form aggregates with varying photo-activities due to their different light scattering properties^46^. Similar effects are expected for the Al NPs whose plasmon resonances are tunable in a broad range from NIR to the deep UV. Plasmonic Al NPs may show photothermal bactericidal activities similar to those of Ag and Au NPs. However, the photothermal inactivation efficiency of the Al NPs is expected to be lower due to their smaller quality factors. Therefore, UV photothermal inactivation and UV protection are two competing effects in plasmonic Al NPs. Our results show the overall increased UV protection of *E*. *coli* by the Al NPs. The possibility of UV protection is a step towards developing new approaches for the controlled bacterial inactivation.

Our results show that Al NPs interact with *E*. *coli* to form aggregates that shield the bacteria from the harmful UV radiation. Because *E*. *coli* bacterial cells have a negative surface charge on the outer membrane^47, 48^, they can electrostatically attach to metallic nanoparticles. Since the Al NPs are efficient light absorbers and scatterers in the UV range (Fig. 3b)^33, 34^, they operate as nanoscale mirrors and attenuators, shielding the attached bacteria from the damaging UV radiation (Figs 1 and 5). We observed UVC shielding capabilities of Al NPs smaller than 2 µm in size (Fig. 3a); whereas, other reports of UVC shielding using non-plasmonic materials were on the order of 20 µm and larger^31, 32^. The enhanced shielding efficiency may be due to the UV plasmonic resonances with enhanced extinction cross sections. In the future, the light scattering properties and UV shielding efficiencies of the Al NPs may be enhanced by the nanoparticle shape and by the thickness of the thin Al_2_O_3_ layer^34, 49^ which is ubiquitously present in the aluminum foil under ambient conditions.

Bacterial cell survival behavior under exposure to radiation can be described using the multi-target or linear-quadratic models of the dose-response curves^50^. Both of these models exhibit a first, shoulder region, characterized by an initially gentle slope for low UV dosage, followed by a second region of steeper decent for higher UV dosage. Our data show similar trends which are modified by the addition of the Al NPs to the *E*. *coli* solution substantially reducing the slope of the second region (Fig. 6c and d). This is known as “tailing” and has been used as an indicator of the protection of the bacterial cells from the UV radiation by association with micrometer size particles^31, 51^. Fractionation of the total dose could possibly provide a further reduction of the slope and more efficient tailing. Here we achieve effective nanotailing behavior using submicrometer size particles which is advantageous in terms of reducing the amount of the protection materials. It may also be used for the UV nanoshielding of viruses^51^ which are an order of magnitude smaller in size than bacteria. The nanotailing effect may be used to control the amount of the UV dosage for the selective inactivation of bacteria and protection of bacteriophages, which may result in more efficient synergistic bacterial inactivation methods.

Another promising future extension of this work may involve application of the Al NPs for the simultaneous UV nanoshielding and surface-enhanced UV resonance biosensing using fluorescence or Raman spectroscopies. Fluorescence was previously used for bio-imaging^37, 52^. Resonant Raman spectroscopy in the UV range was used for the enhancement of weak Raman signals from biomolecules such as proteins^53, 54^ and for cell imaging^55^. Surface-enhanced resonance Raman spectroscopy (SERRS) was performed using aluminum nanoparticles and substrates having UV plasmonic resonances^39^. However, UV radiation used in these spectroscopies was often found damaging to the biological samples located on the surface. UV protection techniques based on energy transfer to lanthanide ions were developed for deep-UV biological imaging^55^. Our method provides an alternative strategy for the simultaneous UV shielding and biosensing/bio-imaging applications which will be explored in the future.

The plasmonic behavior of Al NPs may be tuned to optimize the desired performance. For example, the two competing mechanisms discussed above could be enhanced by increasing either scattering or absorption cross sections of the Al NPs. This will allow for controlling the UV shielding efficiency using various techniques which were developed for designing the plasmonic substrates based on Ag and Au. For example, the arrays of plasmonic nanostructures with desired properties can be fabricated^56–60^. Hot electron generation in Al nanocrystals may provide an alternative mechanism of the bacterial inactivation and will be investigated in the future^61^. Another inactivation mechanism may be realized using aluminum based nanoparticles with photoluminescence properties in the UV range^62^. The bacterial inactivation efficiency may be enhanced by combining these nanoparticles with plasmonic nanostructures. The irregular morphology often results from the spontaneous aggregation of the NPs and influences their plasmonic behavior. In some cases, the irregular or fractal NP aggregates of Ag and Au show strong field enhancement^63–65^. Similar fractal plasmonics behavior may be expected from the Al NP aggregates which may influence the UV nanoshielding efficiency.

Cell toxicity was previously shown to be an important issue in the nanoparticle-based phototherapy^6, 13^ and UV protection^66, 67^. Here, Al NPs are non-toxic to *E*. *coli* bacteria and are similarly expected to be non-toxic to other types of biological cells. They may be functionalized with antibodies for the selective attachment to specific cells and may be used in combination with Ag or Au NPs which may be functionalized for the attachment to other types of cells. This may provide a possibility for the selective inactivation of specific pathogens without damaging other beneficial bacteria or tissue.

UV nanoshielding may also be extended to the ultrashort laser pulse regime. We previously demonstrated single shot detection of bacterial spores using femtosecond adaptive spectroscopic techniques for coherent anti-Stokes Raman scattering (FAST CARS)^68, 69^. We also combined coherent Raman spectroscopy with the plasmonic surface enhancement in time-resolved surface-enhanced CARS (SECARS) spectroscopy using Au nanoparticles^70^. The present results may be used to extend FAST CARS and SECARS to the UV range for ultrasensitive detection and simultaneous UV protection of biomolecules. Al NPs may also be used for developing new types of sunscreen and cosmetics. Our work opens new opportunities for applications of the UV aluminum plasmonics to quantum biophotonics^71^.
